# Genotypic variation in nitrogen use efficiency and yield stability of 28 indica hybrid rice cultivars under tropical nitrogen gradients

**DOI:** 10.3389/fpls.2026.1794288

**Published:** 2026-03-31

**Authors:** Qiuyun Lin, Xi Liu, Yunlu Tian, Zhenyu Xie, Yujie Zhou, Ling Jiang, Wei Hu

**Affiliations:** 1 Tropical Crops Genetic Resources Institute, Chinese Academy of Tropical Agricultural Sciences, Haikou, China; 2 Key Laboratory of Crop Gene Resources and Germplasm Enhancement in Southern China, Ministry of Agriculture and Rural Affairs, Haikou, China; 3 Key Laboratory of Tropical Crops Germplasm Resources Genetic Improvement and Innovation of Hainan Province, Haikou, China; 4 State Key Laboratory for Crop Genetics and Germplasm Enhancement, Jiangsu Nanjing National Field Scientific Observation and Research Station for Rice Germplasm, Nanjing Agricultural University, Nanjing, China

**Keywords:** indica hybrid rice, nitrogen agronomic efficiency, nitrogen response pattern, rice yield, tropical agroecosystem

## Abstract

**Introduction:**

Excessive nitrogen (N) application is a prevalent issue in tropical rice production, often leading to environmental degradation without guaranteeing yield gains. Exploiting genotypic potential for low-N tolerance is essential for sustainable intensification.

**Methods:**

This study investigated the N response of 28 indica hybrid rice cultivars under a gradient of N rates (60–240 kg N ha^−^¹) in the tropical climate of Hainan, China.

**Results:**

We identified significant genotypic divergence in yield sensitivity to N, categorizing the varieties into four functional groups: High-efficiency/High response, Low-efficiency/High-response, High-efficiency/Low-response, and High-N inhibited. The differentiation in yield potential was mechanistically linked to plasticity in effective panicle number and sink size (spikelet number per panicle). Crucially, this study highlights a pathway for N reduction: 10 identified cultivars maintained yields exceeding 8.0 t ha^−^¹ at a medium-low N rate (120 kg N ha^−^¹), achieving a 33% reduction in N fertilizer usage compared to the local standard (180 kg N ha^−^¹) without yield compromise.

**Discusssion:**

These “N-efficient and stable-yielding” varieties demonstrated superior N agronomic efficiency, suggesting that optimizing variety selection can reduce rice productivity’s dependence on high N inputs. These results provide scientific support for N reduction and efficiency-enhancement strategies in tropical agroecosystems.

## 1 Introduction

Nitrogen (N) is the most limiting nutrient for cereal crops and plays a decisive role in global food security. For rice (*Oryza sativa* L.), which feeds over half of the world’s population, adequate N supply is essential for optimizing canopy photosynthesis, biomass accumulation, and yield formation (Liao et al., 2019; Bhardwaj et al., 2022). However, the pursuit of higher yields has led to the excessive application of N fertilizers in many intensive rice-producing regions, particularly in Asia (Zhang et al., 2020; Hu et al., 2023). This “insurance” approach to fertilization not only reduces nitrogen use efficiency (NUE) but also triggers severe environmental cascades, including soil acidification, water eutrophication, and elevated greenhouse gas emissions (Muthayya et al., 2014; Neeraja et al., 2021). Among the various indices used to evaluate NUE, nitrogen agronomic efficiency (NAE)—defined as the increase in grain yield per unit of applied nitrogen—is widely adopted as a practical field-level indicator of fertilizer-use effectiveness (Barłóg, 2023). Consequently, a major challenge for modern agriculture is to decouple grain yield maximization from heavy nitrogen dependence (Liu et al., 2021).

The response of rice grain yield to N input is governed by a complex interaction between genotype, environment, and management (Good et al., 2004). While conventional high-yielding varieties often rely on high N inputs to realize their potential, this strategy is increasingly unsustainable (Haefele et al., 2008). Crucially, significant genotypic variation exists in N uptake and utilization efficiency among rice cultivars (Ishikawa et al., 2005; Liu et al., 2023). Indica hybrid rice, which dominates rice production in China due to its significant heterosis, generally exhibits higher yield potential and N demand compared to inbred lines. However, hybrid cultivars vary widely in their sensitivity to N levels; some are “N-hungry, “ requiring high inputs, while others are “N-efficient, “ maintaining high productivity under reduced N conditions (Wing et al., 2018; Gao et al., 2022; Liu et al., 2022a). Exploiting this genetic variability is therefore a promising pathway to identify cultivars that can tolerate reduced N inputs without yield penalties.

Although numerous studies have screened N-efficient cultivars in temperate and subtropical regions, rice production in tropical regions faces distinct agro-ecological challenges that remain under-investigated. Hainan Province, the southernmost tropical rice-growing region in China, is characterized by abundant solar radiation and high temperatures. These conditions accelerate crop development, significantly shortening the vegetative growth phase and grain-filling duration compared to temperate zones. Furthermore, high rainfall in tropical climates (Xie et al., 2025) exacerbates N leaching, complicating fertilizer management. Consequently, high-yielding cultivars selected in other regions may not perform optimally in Hainan’s unique hydrothermal environment. Few studies have evaluated N-response plasticity of indica hybrids under tropical conditions characterized by high leaching risk and accelerated phenology. How diverse indica hybrid rice genotypes adapt to varying N levels under these specific tropical constraints, particularly in the context of N reduction strategies, remains poorly understood.

Unlike previous NUE screening studies that primarily used discrete nitrogen levels and focused mainly on yield potential, this study evaluates hybrid indica–specific responses across a four-level N gradient under tropical conditions. Moreover, we emphasize yield stability under reduced nitrogen input, rather than high-yield performance alone, to better inform sustainable nitrogen management strategies. To address this gap, identifying cultivars with superior phenotypic plasticity—those that maintain high yield stability under low to moderate N inputs—is essential for the region’s sustainable development. Therefore, this study conducted a comprehensive field experiment in Lingshui, Hainan, evaluating 28 indica hybrid rice varieties under four N gradients. The specific objectives were to: (1) evaluate the agronomic performance and yield components of diverse cultivars to reveal their specific N-response patterns; (2) quantify the Nitrogen Agronomic Efficiency to differentiate genotype efficiency; and (3) identify elite cultivars capable of maintaining stable high yields under reduced N conditions. This study aims to provide a scientific basis for breeding N-efficient varieties adapted to tropical climates and to offer practical guidance for optimizing N management, thereby facilitating a “low-input, high-output” rice production system in tropical agroecosystems.

## 2 Materials and methods

### 2.1 Experimental site and plant materials

The field experiment was conducted during the 2024 rice growing season at the Research Base of Hainan Guangling Investment Management Group Co., Ltd., located in Qinfeng Village, Lingshui County, Hainan Province, China (18°29′15″ N, 110°1′46″ E). The region is characterized by a typical tropical monsoon climate with abundant rainfall and solar radiation. The material growth period spanned from February to June 2024, during which the monthly average low temperatures ranged from 19 °C to 25 °C, and the monthly average high temperatures ranged from 27 °C to 33 °C. The extreme high temperatures in February, March, April, May, and June were 30 °C, 33 °C, 34 °C, 35 °C, and 35 °C, respectively. The cumulative precipitation totaled 168.7 mm, with 99.8% occurring in May and June, reflecting the “high temperature coinciding with rainy season” pattern characteristic of a tropical monsoon climate. The experimental site was intentionally selected in a tropical rice-growing region because tropical production systems are characterized by concentrated rainfall and high temperatures, which can accelerate crop phenology and alter nitrogen (N) dynamics (e.g., increasing the risk of rainfall-driven N losses). These features may lead to nitrogen-response patterns that differ from those observed in conventional temperate/subtropical rice regions. Therefore, a region-specific field evaluation under tropical conditions is necessary to screen cultivar performance and yield stability across N gradients.

The soil at the experimental site is classified as a sandy loam (locally known as “black sand soil”). Pre-experiment soil sampling (0–20 cm depth) indicated the following physicochemical properties: total nitrogen 2.43 g kg^−^¹, total phosphorus 546.83 mg kg^−^¹, total potassium 4.26 g kg^−^¹, available nitrogen (alkali-hydrolyzable N) 56.47 mg kg^−^¹, available phosphorus (Olsen-P) 45.78 mg kg^−^¹, and available potassium 44.99 mg kg^−^¹.

Twenty-eight indica hybrid rice cultivars, originating from diverse ecological zones across China (including Jiangsu, Zhejiang, Anhui, Hunan, and Guangxi provinces), were selected for this study. These cultivars represent the dominant high-yielding genetic backgrounds currently utilized in southern China (**Table 1**). The selection criteria included production relevance, seed availability, and demonstrated adaptation to southern rice-growing environments. The use of a diverse and production-relevant panel enhances the robustness of the subsequent classification and its applicability to nitrogen-reduction strategies in tropical rice systems.

**Table 1:** Information on the 28 indica hybrid rice cultivars used in this study.

ID	Cultivar name	Breeding institutions	Origin (Province)
V01	Huangyou 172	Hunan Shenzhou Xingrui Seed Industry Co., Ltd.	Hunan
V02	Mingliangyou 896	Jiangsu Mingtian Seed Industry Technology Co., Ltd.	Jiangsu
V03	Yingliangyou Mingyuesimiao	Jiangsu Mingtian Seed Industry Technology Co., Ltd.	Jiangsu
V04	Yingliangyouxiang 28	Jiangsu Mingtian Seed Industry Technology Co., Ltd.	Jiangsu
V05	Huazheyou 901	China National Rice Research Institute (CNRRI)	Zhejiang
V06	Zhongzheyou 196	Zhejiang Wufangnong Seed Industry Co., Ltd.; Hunan Changde Fengyu Seed Co., Ltd.	Zhejiang
V07	Huazheyou 210	China National Rice Research Institute (CNRRI); Zhejiang Wufangnong Seed Industry Co., Ltd.	Zhejiang
V08	Zhejingyou 27	Zhejiang Wufangnong Seed Industry Co., Ltd.; China National Rice Research Institute (CNRRI); Institute of Crop and Nuclear Technology Utilization, Zhejiang Academy of Agricultural Sciences	Zhejiang
V09	Xinliangyouxiang 66	Anhui Quanyin High-Tech Seed Industry Co., Ltd.	Anhui
V10	Kangxiangyou 135	Yuan Longping Agricultural High-Tech Co., Ltd. (Longping High-Tech); Guangxi Hengmao Agricultural Science and Technology Co., Ltd.; Jiangxi Keyuan Seed Industry Co., Ltd.	Hunan
V11	Zhenyou 13 Xiang	Guangxi Hengmao Agricultural Science and Technology Co., Ltd.; Guangxi Baixiang High-Tech Seed Industry Co., Ltd.	Guangxi
V12	Shouxiangliangyouxiang 99	Guangxi Hengmao Agricultural Science and Technology Co., Ltd.	Guangxi
V13	Youxiangyou Lixiang 20	Guangxi Zhuangbang Seed Industry Co., Ltd.; Guangxi Zhaohe Seed Industry Co., Ltd.	Guangxi
V14	Bangliangyou 15 Xiang	Guangxi Zhaohe Seed Industry Co., Ltd.; Guangxi Zhuangbang Seed Industry Co., Ltd.	Guangxi
V15	Youxiangyou Xiangsimiao	Guangxi Zhaohe Seed Industry Co., Ltd.; Guangxi Zhuangbang Seed Industry Co., Ltd.	Guangxi
V16	Quanyou 836	Anhui Quanyin High-Tech Seed Industry Co., Ltd.	Anhui
V17	Quanliangyou 6019	Anhui Quanyin High-Tech Seed Industry Co., Ltd.	Anhui
V18	Quanyou 392	Anhui Quanyin High-Tech Seed Industry Co., Ltd.	Anhui
V19	Quanyou 879	Anhui Quanyin High-Tech Seed Industry Co., Ltd.; Guangxi Quanhong Agricultural Science and Technology Co., Ltd.	Anhui
V20	Yangxianyou 986	Nanjing Agricultural University	Jiangsu
V21	Yangxianyou 906	Nanjing Agricultural University	Jiangsu
V22	Nan 9 You 801	Nanjing Agricultural University	Jiangsu
V23	Nanxian 29023	Jiangsu Academy of Agricultural Sciences	Jiangsu
V24	Quanliangyou 82 Xiang	Anhui Quanyin High-Tech Seed Industry Co., Ltd.	Anhui
V25	Quanyou 535	Anhui Quanyin High-Tech Seed Industry Co., Ltd.	Anhui
V26	Quanyou Hongmeng	Anhui Quanyin High-Tech Seed Industry Co., Ltd.	Anhui
V27	Quanliangyou 686	Anhui Quanyin High-Tech Seed Industry Co., Ltd.	Anhui
V28	Yinliangyou Jingzhan	Anhui Quanyin High-Tech Seed Industry Co., Ltd.	Anhui

### 2.2 Experimental design and field management

The study was arranged in a split-plot design with three replicates. The main plots were assigned to nitrogen (N) treatments, while the subplots were assigned to the 28 rice cultivars. Four nitrogen application rates were established: 60 kg N ha^−^¹ (Low Nitrogen, LN), 120 kg N ha^−^¹ (Medium-Low Nitrogen, MLN), 180 kg N ha^−^¹ (Normal Nitrogen, NN), and 240 kg N ha^−^¹ (High Nitrogen, HN). Additionally, a zero-N control (N0) was included for all cultivars to calculate NAE. The rate of 180 kg N ha^−^¹ (NN) represents the prevailing local farmer practice for hybrid rice production in the region. The low nitrogen rate of 60 kg N ha^−^¹ (LN) was set as a reduced-input baseline to evaluate cultivar performance under nitrogen-limited conditions. The high nitrogen rate of 240 kg N ha^−^¹ (HN) was included to represent excessive fertilizer input occasionally observed in intensive systems and to assess potential yield inhibition and nitrogen-use inefficiency under high N supply. To prevent water and nutrient lateral seepage, each plot was separated by rigid plastic barriers (50 cm width) inserted 20 cm deep into the soil. Each subplot covered an area of 3.0 m² (1.5 m × 2.0 m).

Seeds were sown in plastic trays on February 15, 2024. On March 12, 25-day-old seedlings were manually transplanted into the field at a spacing of 25 cm × 15 cm, with one seedling per hill, simulating mechanical transplanting density. Nitrogen fertilizer (urea) was applied in three splits: 40% as basal fertilizer (1 day before transplanting), 30% at the tillering stage (7 days after transplanting), and 30% at the panicle initiation stage. To ensure that N was the only limiting factor, phosphorus (375 kg superphosphate with 12% P_2_O_5_ ha^-1^) and potassium (90 kg potassium chloride with 60% K_2_O ha^−1^) were all applied as basal fertilizers across all plots 2 days before transplanting in each treatment. Other agronomic practices, including irrigation and pest management, were performed in accordance with local high-yield cultivation standards.

### 2.3 Sampling, measurements, and calculations

At the physiological maturity stage, plant height and the number of effective panicles were determined from a representative row in each plot to calculate the plot mean. To minimize border effects and sampling bias associated with small plot size, three representative hills with effective panicle numbers close to the plot mean were sampled for yield component analysis. The number of effective panicles, spikelets per panicle, filled grain percentage, and 1000-grain weight were measured using an automatic seed testing instrument (Wanshen Detection Technology Co., Ltd., Hangzhou, China). Grain yield was calculated based on the measured yield components and plant density (hills per unit area) and adjusted to a standard moisture content of 14%.

Nitrogen use efficiency was evaluated using NAE, which represents the yield increase per unit of applied N. Among the various NUE indices, NAE was selected because it serves as a practical field-level indicator that directly reflects the agronomic response of grain yield to nitrogen input, without requiring measurements of plant nitrogen uptake. NAE (kg kg^-1^) was calculated using the following equation:


$$NAE=\frac{Y_{N_{A}}-Y_{N_{0}}}{N_{A}}$$

Where Y_NA_ and Y_N0_ are the yield of the plots with and without nitrogen applications (kg ha^-1^), respectively; and N_A_ is the amount of nitrogen applied (kg N ha^-1^).

### 2.4 Statistical analysis

Data compilation and preliminary processing were performed using Microsoft Excel 2019. Statistical analyses were conducted using SPSS 27.0 (IBM Corp., Armonk, NY, USA). A two-way analysis of variance (ANOVA) was employed to evaluate the effects of Cultivar (V), Nitrogen rate (N), and their interaction (V × N) on grain yield and yield components. When F-tests were significant, means were compared using the Least Significant Difference (LSD) test at probability levels of *P* < 0.05 and *P* < 0.01. Cluster analysis was performed to manually classify 28 varieties based on their yield responses under Medium-Low and high nitrogen levels. All figures were generated using Origin 2022 (OriginLab Corp., Northampton, MA, USA).

## 3 Results

### 3.1 Analysis of variance and general yield response

**Figure 1:**
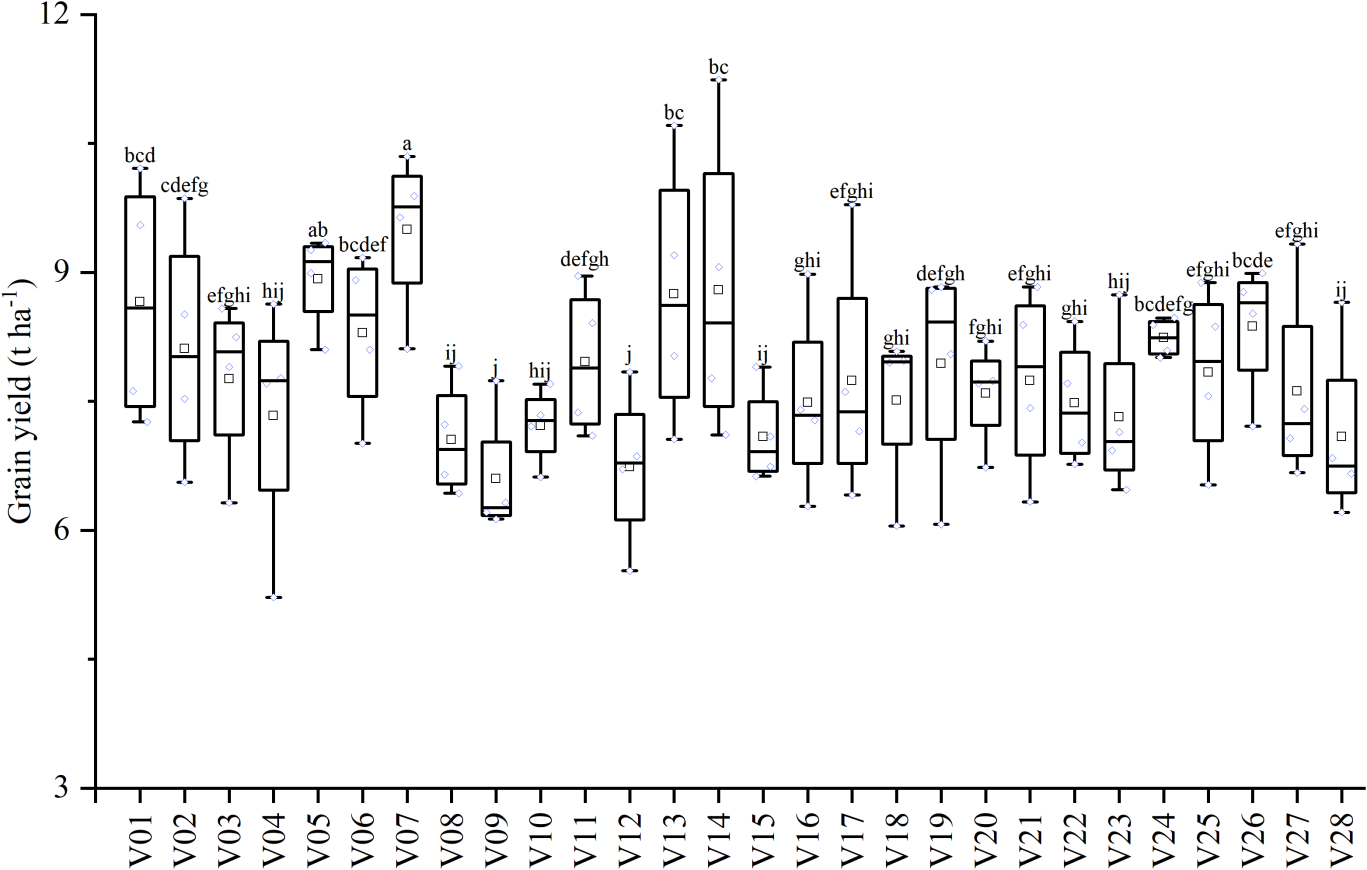
Grain yield performance of 28 hybrid rice varieties under four nitrogen application rates. The nitrogen rates are LN (60 kg N ha^−^¹), MLN (120 kg N ha^−^¹), NN (180 kg N ha^−^¹), and HN (240 kg N ha^−^¹). Different lowercase letters above the bars indicate significant differences among varieties average grain yield across the four nitrogen application rates at *P* < 0.05.

The analysis of variance (ANOVA) revealed highly significant effects (*P* < 0.01) of genotype (V) and nitrogen application rate (N) on grain yield and all measured yield components, with the exception of the effect of N rate on filled grain percentage, which was significant at *P* < 0.05 (**Table 2**). The interaction effect between genotype and nitrogen rate (V × N) was highly significant for grain yield, effective panicles per hill, filled grain percentage, and 1000-grain weight (*P* < 0.01), but not for spikelet number per panicle (P > 0.05). This significant V × N interaction indicates that the 28 hybrid rice cultivars responded differentially to increasing N inputs. Across all treatments, grain yield varied substantially among cultivars and N levels (**Figure 1**). Cultivar V07 exhibited the highest average yield (9.49 ± 0.97 t ha^−^¹) across the four N gradients, demonstrating superior yield potential. Conversely, V09 had the lowest average yield (6.60 ± 0.77 t ha^−^¹). The sensitivity to N input also differed markedly. For instance, V14 showed high N sensitivity, with a yield increase of 58.02% (4.13 t ha^−^¹) from the low nitrogen (LN) to the high nitrogen (HN) treatment. In contrast, V24 displayed strong yield stability, with a fluctuation of only 0.46 t ha^−^¹ between its highest and lowest yields, maintaining a high average yield of 8.24 ± 0.22 t ha^−^¹ across all N levels (**Table 3**). Although SNP of V24 decrease from 184.91 (LN) to 158.84 (MLN), compensatory increases in EP may explain yield stability (**Table 3**).

**Table 2:** Analysis of variance (F-values) for grain yield and yield components as affected by variety and nitrogen application rate.

ANOVA	GY	EP	SNP	FGP	GW
Variety (V)	9.128^**^	39.391^**^	17.542^**^	17.482^**^	349.533^**^
Nitrogen application rate (N)	93.883^**^	69.201^**^	10.634^**^	2.676^*^	16.902^**^
V × N	2.129^**^	1.817^**^	1.31^ns^	2.532^**^	1.68^**^

GY, Grain yield; EP, Effective panicles per hill; SNP, Spikelet number per panicle; FGP, Filled grain percentage; GW, 1000-grain weight. V, Variety; N, Nitrogen application rate. ^*^ and ^**^ denote statistical significance at *P* < 0.05 and *P* < 0.01, respectively; ns indicates non-significance.

**Table 3:** Grain yield and yield components of representative rice cultivars under different nitrogen treatments in the 2024 growing season.

Variety	Nitrogen treatment (kg N ha^-1^)	GY (t ha^− 1^)	EP	SNP	FGP (%)	GW (g)
V07	60 (LN)	8.11 ± 0.49^b^	8.00 ± 0.36^a^	188.31 ± 7.67^a^	81.16 ± 1.80^a^	20.11 ± 0.68^a^
	120 (MLN)	9.89 ± 0.33^a^	9.48 ± 1.14^a^	199.39 ± 7.59^a^	77.98 ± 4.74^a^	20.45 ± 0.29^a^
	180 (NN)	9.64 ± 0.43^a^	9.23 ± 1.05^a^	205.43 ± 6.23^a^	78.54 ± 1.29^a^	19.74 ± 0.43^a^
	240 (HN)	10.35 ± 0.49^a^	10.00 ± 0.82^a^	197.81 ± 10.25^a^	78.48 ± 3.09^a^	20.31 ± 0.96^a^
V09	60 (LN)	6.13 ± 0.93^a^	7.18 ± 0.42^b^	152.93 ± 29.68^a^	78.91 ± 3.13^a^	21.60 ± 0.40^a^
	120 (MLN)	6.21 ± 1.67^a^	7.91 ± 0.90^b^	160.17 ± 14.22^a^	68.86 ± 9.22^a^	21.30 ± 0.87^a^
	180 (NN)	6.32 ± 1.23^a^	8.52 ± 0.58^ab^	143.78 ± 11.89^a^	70.00 ± 6.31^a^	22.22 ± 1.27^a^
	240 (HN)	7.74 ± 1.69^a^	9.72 ± 1.21^a^	149.28 ± 18.01^a^	73.42 ± 5.59^a^	22.14 ± 0.37^a^
V14	60 (LN)	7.11 ± 0.49^c^	8.78 ± 0.19^b^	183.97 ± 9.41^a^	65.86 ± 4.30^a^	20.28 ± 0.44^a^
	120 (MLN)	7.77 ± 1.04^bc^	10.33 ± 1.53^ab^	179.37 ± 17.33^a^	61.90 ± 5.16^a^	20.67 ± 0.36^a^
	180 (NN)	9.06 ± 0.55^b^	10.89 ± 0.69^a^	190.81 ± 1.99^a^	64.15 ± 3.97^a^	20.62 ± 0.70^a^
	240 (HN)	11.24 ± 0.58^a^	11.33 ± 0.67^a^	196.09 ± 12.49^a^	72.50 ± 3.42^a^	21.19 ± 0.07^a^
V24	60 (LN)	8.09 ± 0.38^a^	7.21 ± 0.74^a^	184.91 ± 23.91^a^	83.42 ± 3.08^a^	22.23 ± 0.20^a^
	120 (MLN)	8.01 ± 0.84^a^	8.64 ± 0.88^a^	158.84 ± 8.22^a^	78.09 ± 6.94^a^	22.76 ± 0.99^a^
	180 (NN)	8.47 ± 0.13^a^	7.95 ± 0.59^a^	184.82 ± 4.92^a^	78.00 ± 3.70^a^	22.49 ± 0.35^a^
	240 (HN)	8.39 ± 0.49^a^	7.91 ± 0.40^a^	192.97 ± 7.64^a^	74.17 ± 4.36^a^	22.51 ± 0.6^a^

GY, Grain yield; EP, Effective panicles per hill; SNP, Spikelet number per panicle; FGP, Filled grain percentage; GW, 1000-grain weight. LN, Low Nitrogen (60 kg N ha^−^¹); MLN, Medium-Low Nitrogen (120 kg N ha^−^¹); NN, Normal Nitrogen (180 kg N ha^−^¹); HN, High Nitrogen (240 kg N ha^−^¹). Data represent means of three replicates. Different lowercase letters within the same column for each variety indicate significant differences among nitrogen treatments at *P* < 0.05 according to the LSD test.

### 3.2 Classification of cultivars based on nitrogen response patterns

**Figure 2:**
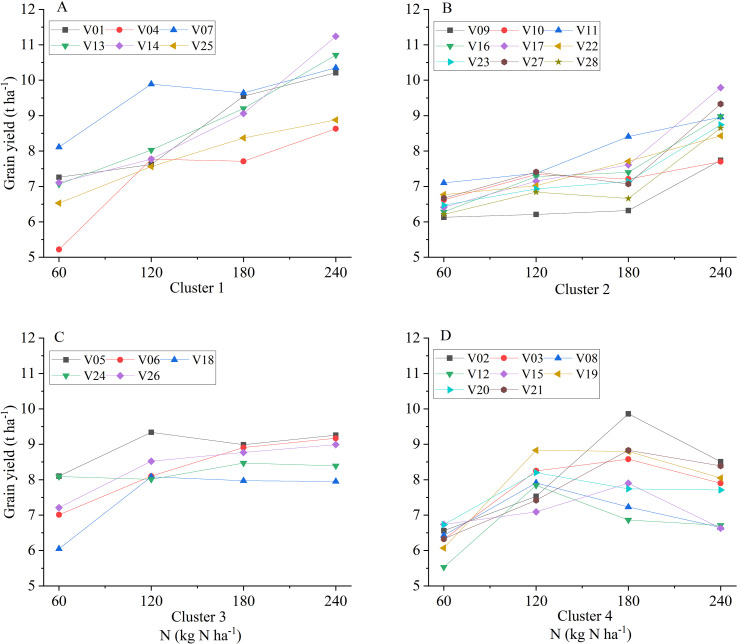
Classification of 28 hybrid rice varieties into four clusters based on based on yield responses at LN, MLN, NN, and HN. **(A)** Cluster 1: High Potential Type (Efficient and Responsive); **(B)** Cluster 2: Late Responder Type (Inefficient but Responsive); **(C)** Cluster 3: Stable Type (Efficient and Tolerant); **(D)** Cluster 4: Inhibition Type (Sensitive to High N).

**Figure 3:**
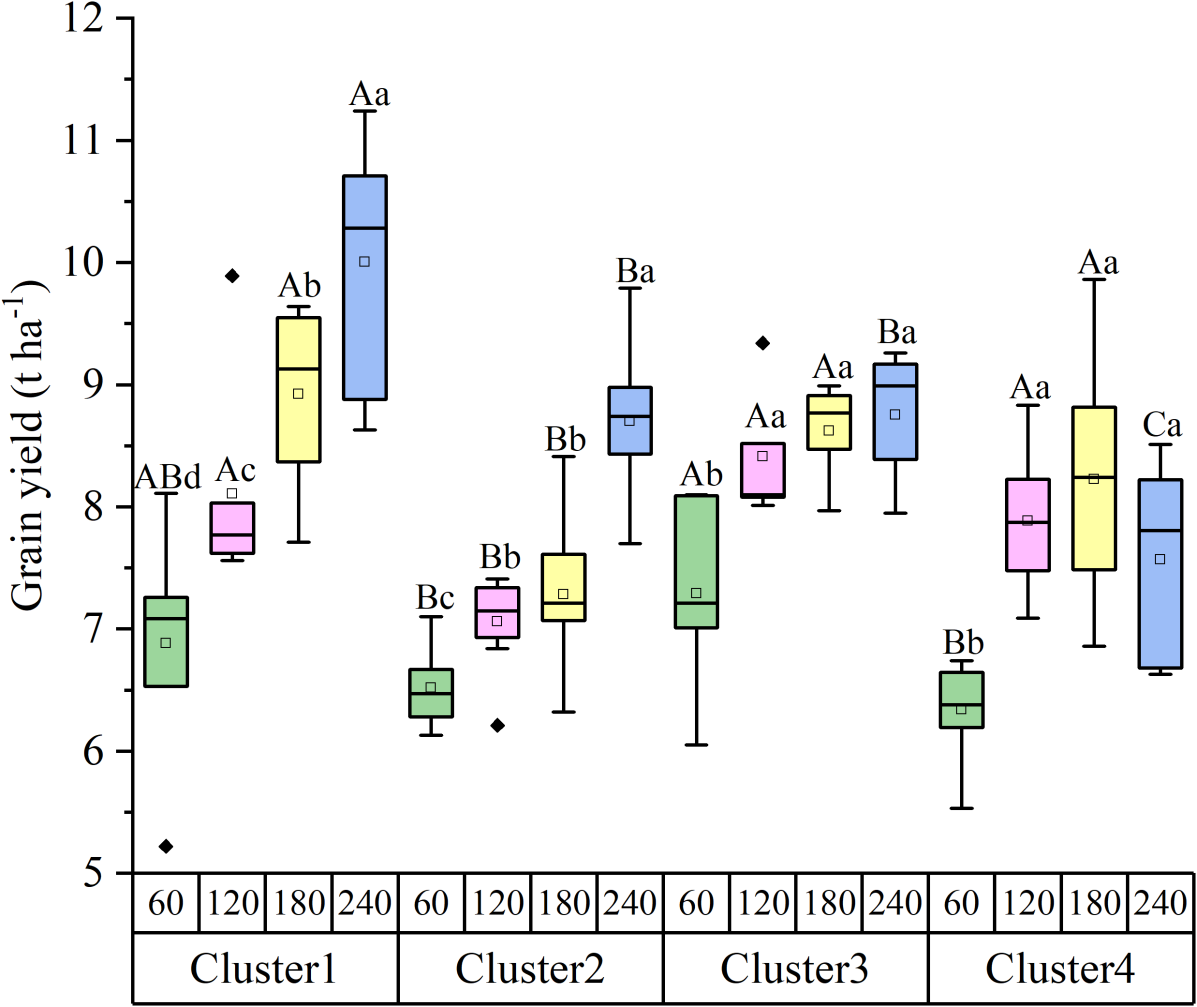
Grain yield of the four nitrogen-responsive clusters under different nitrogen application rates. Cluster 1, High Potential Type; Cluster 2, Late Responder Type; Cluster 3, Stable Type; Cluster 4, Inhibition Type. Different lowercase letters indicate significant differences among nitrogen rates within the same cluster (*P* < 0.05). Different capital letters indicate significant differences among clusters at the same nitrogen rate (*P* < 0.05).

Based on the yield response curves to increasing N application rates, the 28 cultivars were categorized into four distinct clusters (**Figures 2**, **3**). Cluster 1 (High Potential Type) comprised six cultivars (V01, V04, V07, V13, V14, V25) characterized by high yields (>7.5 t ha^−^¹) at the medium-low nitrogen (MLN) level and continuous yield increases with higher N inputs. For example, V13 achieved 8.03 t ha^−^¹ at MLN and further increased its yield by 30.26% and 51.57% at normal nitrogen (NN) and HN levels, respectively, compared to the LN baseline. Cluster 2 (Late Responder Type) included nine cultivars (V09, V10, V11, V16, V17, V22, V23, V27, V28) that performed poorly (<7.5 t ha^−^¹) at low to medium N inputs but showed a sharp yield increase only at the highest N level (240 kg N ha^−^¹). For instance, V17 produced only 7.15 t ha^−^¹ at MLN but jumped to 9.79 t ha^−^¹ at HN, a 52.62% increase over the LN yield. Cluster 3 (Stable Type) consisted of five cultivars (V05, V06, V18, V24, V26) that achieved high yields (> 8.0 t ha^−^¹) at the MLN level, with yield gains plateauing upon further N additions. V26, for example, reached 8.52 t ha^−^¹ at 120 kg N ha^−^¹, with no significant yield benefit at higher N rates. Finally, Cluster 4 (Inhibition Type) comprised eight cultivars (V02, V03, V08, V12, V15, V19, V20, V21). Yields in this group increased initially from LN to NN but declined significantly (by 5.0%–15.95%) at the HN level, indicating poor tolerance to excessive nitrogen. V02, for instance, peaked at the NN level but decreased by 13.71% at HN.

The average yield performance differed significantly among clusters across N levels (**Figure 3**). Under LN conditions (60 kg N ha^−^¹), Cluster 3 exhibited the highest average yield (7.29 ± 0.85 t ha^−^¹), significantly outperforming other clusters (*P* < 0.05). Under MLN conditions (120 kg N ha^−^¹), Cluster 2 had the lowest average yield (7.06 ± 0.38 t ha^−^¹), while Clusters 1, 3, and 4 all exceeded 7.5 t ha^−^¹, showing increases of 14.80%, 19.13%, and 11.67% over Cluster 2, respectively. At the HN level (240 kg N ha^−^¹), Cluster 1 achieved the highest average yield (10.00 ± 1.03 t ha^−^¹), whereas Cluster 4 showed a declining trend, dropping to 7.57 ± 0.79 t ha^−^¹.

### 3.3 Response of yield components to nitrogen among clusters

**Figure 4:**
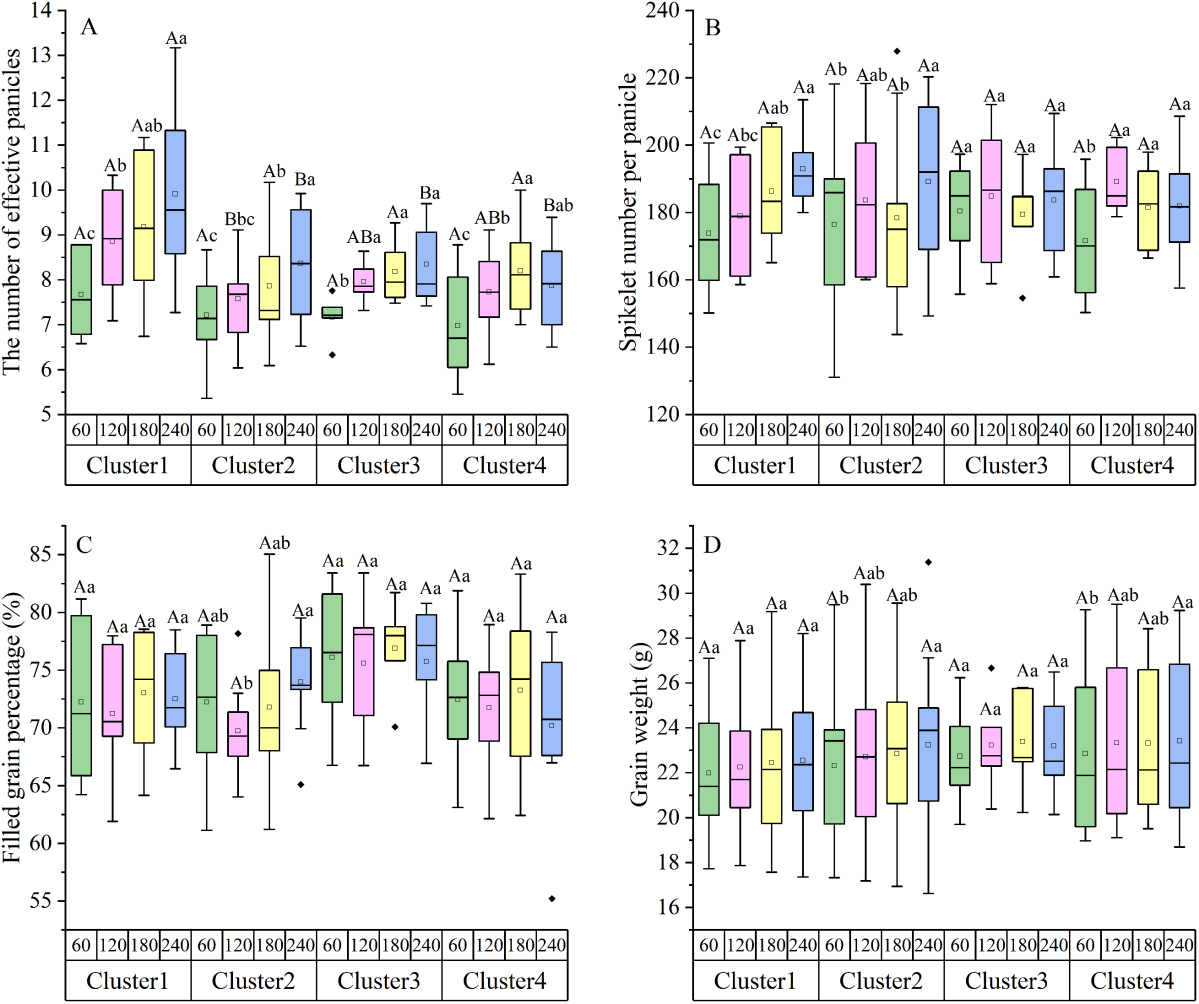
Effects of nitrogen application rates on yield components of the four nitrogen-responsive clusters. **(A)** Effective panicles per hill; **(B)** Spikelet number per panicle; **(C)** Filled grain percentage; **(D)** 1000-grain weight. Different lowercase letters indicate significant differences among nitrogen rates within the same cluster (*P<* 0.05). Different capital letters indicate significant differences among clusters at the same nitrogen rate (*P<* 0.05).

The variation in grain yield among clusters was primarily driven by the differential responses of effective panicles per hill (EP) and spikelet number per panicle (SNP) to N application (**Figure 4**). For Cluster 1, EP increased significantly by 15.49%, 19.65%, and 29.19% at MLN, NN, and HN levels, respectively, compared to LN (*P* < 0.05). Similarly, SNP increased continuously by 3.00% to 11.04%. This simultaneous increase in both source and sink size underpins their high yield potential at high N. In Cluster 2, the increase in EP was moderate at lower N levels but surged by 16.06% at HN. Notably, SNP showed limited response at MLN and NN levels but increased markedly by 7.19% at the HN level, suggesting that sink size expansion in these varieties requires a high N threshold. For Cluster 3, SNP increased significantly by 10.99% at the MLN level compared to LN (*P* < 0.05). However, further N application (NN and HN) did not lead to additional significant increases in SNP compared to MLN. EP also showed a similar plateauing trend, explaining the yield stability of this group at moderate N levels. In contrast, for Cluster 4, while EP increased with N application, SNP showed a distinct response: it increased by 10.74% and 17.42% at MLN and NN levels, respectively, but declined at the HN level. This reduction in sink size at high N likely contributes to the observed yield penalty in this group. Filled grain percentage and 1000-grain weight showed relatively small variations across N treatments compared to panicle traits (**Figures 4C, D**), indicating that the divergence in N responsiveness among the four clusters is mainly attributed to the plasticity of effective panicle number and spikelet number per panicle.

### 3.4 NAE and correlation analysis

**Figure 5:**
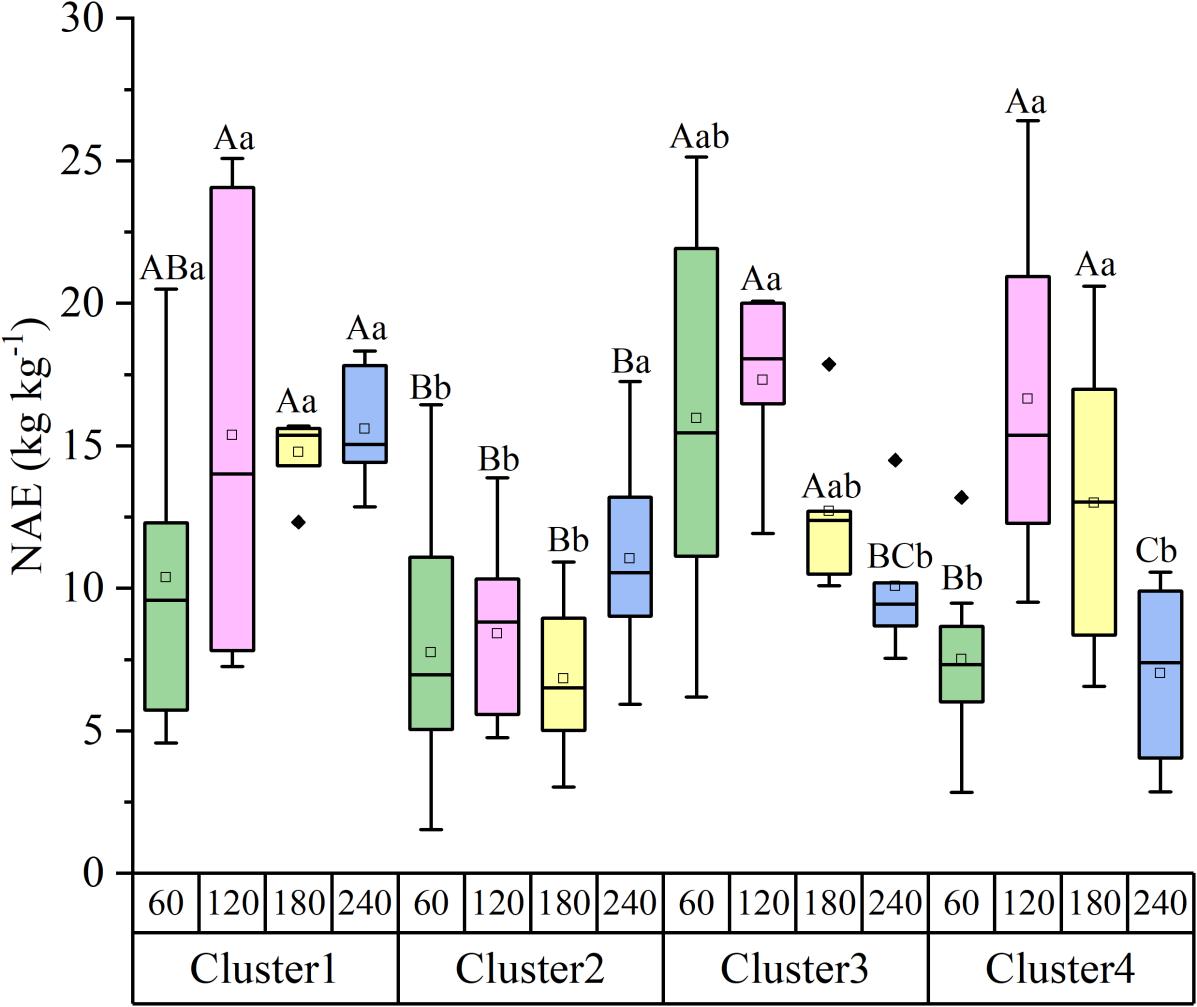
NAE of the four nitrogen-responsive clusters under varying nitrogen application rates. NAE represents the yield increase per kg of applied N (kg kg^−^¹). Different lowercase letters indicate significant differences among nitrogen rates within the same cluster (*P* < 0.05). Different capital letters indicate significant differences among clusters at the same nitrogen rate (*P* < 0.05).

NAE varied significantly among the four clusters across different N levels (**Figure 5**). Cluster 1 maintained consistently high NAE values (10.38 kg kg^-1^, 15.37 kg kg^-1^, 14.78 kg kg^-1^, and 15.58 kg kg^-1^ at LN, MLN, NN, and HN, respectively), confirming its classification as a “high-efficiency, high-response” group. In contrast, Cluster 2 exhibited relatively low NAE at lower N rates (7.74 kg kg^-1^ at LN) but showed improved efficiency at high N (11.05 kg kg^-1^ at HN), consistent with its “low-efficiency, high-response” profile. Cluster 3 maintained high NAE at low to medium N levels (17.30 kg kg^-1^ at MLN) but experienced a sharp decline at the HN level (10.07 kg kg^-1^), aligning with its “high-efficiency, stable” characteristics. Cluster 4 showed a drastic reduction in NAE at the HN level (dropping to 7.01 kg kg^-1^), accompanied by yield loss, further validating its classification as “high-N sensitive.”

Correlation analysis (**Table 4**) further elucidated the physiological basis of yield and NAE. Grain yield was significantly and positively correlated with effective panicle number (*r* = 0.533^**^), spikelet number per panicle (*r* = 0.413^**^), and filled grain percentage (*r* = 0.265^**^). Similarly, NAE showed significant positive correlations with these three traits (*r* = 0.213^*^, 0.227^*^, and 0.317^**^, respectively). However, 1000-grain weight showed no significant correlation with either grain yield or NAE. These results suggest that improving panicle number and spikelet fertility is critical for simultaneously enhancing yield and nitrogen use efficiency in hybrid rice.

**Table 4:** Pearson correlation coefficients between yield components, grain yield, and NAE.

Agronomic traits	Measured parameters			
	EP	SNP	FGP (%)	GW (g)
Grain yield (t ha^-1^)	0.533^**^	0.413^**^	0.265^**^	-0.025
NAE (kg kg^-1^)	0.213^*^	0.227^*^	0.317^**^	-0.051

EP, Effective panicles per hill; SNP, Spikelet number per panicle; FGP, Filled grain percentage; GW, 1000-grain weight. V, Variety; NAE, nitrogen agronomic efficiency. ^*^ and ^**^ denote statistical significance at *P* < 0.05 and *P* < 0.01, respectively.

### 3.5 Identification of cultivars suitable for nitrogen-reduced cultivation

**Figure 6:**
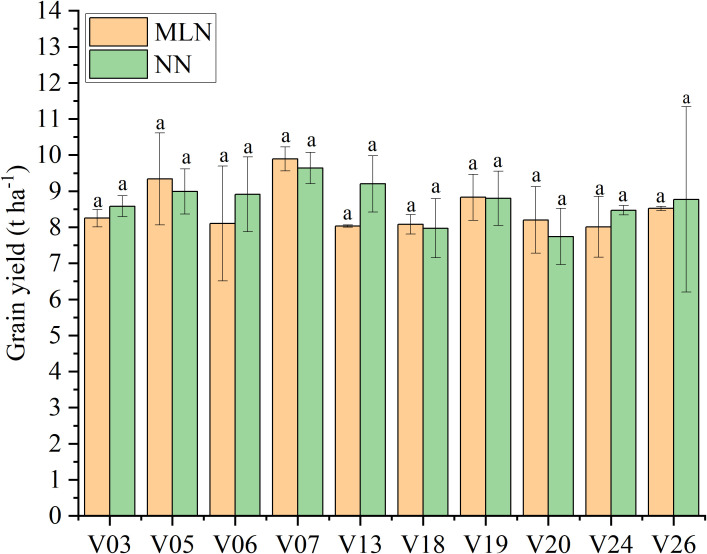
Identification of elite rice varieties maintaining stable high yields under reduced nitrogen conditions. Comparison of grain yields between Medium-Low Nitrogen (120 kg N ha^−^¹) and Normal Nitrogen (180 kg N ha^−^¹) levels. MLN, Medium-Low Nitrogen (120 kg N ha^−^¹); NN, Normal Nitrogen (180 kg N ha^−^¹). Error bars represent the standard error of the mean (n = 3). Different lowercase letters indicate significant differences between the two nitrogen treatments for the same variety at *P* < 0.05.

A key objective of this study was to identify cultivars capable of maintaining high yields under reduced N input. Comparison between the MLN (120 kg N ha^−^¹, a 33% reduction from local farmer practice) and NN (180 kg N ha^−^¹, the regional standard nitrogen application rate) treatments revealed that ten cultivars (V03, V05, V06, V07, V13, V18, V19, V20, V24, and V26) maintained yields exceeding 8.0 t ha^−^¹ under reduced N conditions (**Figure 6**). Notably, cultivars V05 and V07 achieved yields greater than 9.0 t ha^−^¹ even at MLN. Statistical analysis confirmed no significant yield difference between MLN and NN treatments for these cultivars. Furthermore, the NAE at the MLN level remained high, ranging from 11.92 kg kg^-1^ to 26.41 kg kg^-1^. It is noteworthy that five of these elite cultivars (V05, V06, V18, V24, V26) belong to Cluster 3 (Stable Type).

## 4 Discussion

### 4.1 Genotypic variation in yield response patterns and its genetic basis

Although nitrogen reduction studies are frequently conducted using conventional japonica rice (Meng et al., 2021; Yong and Zhang, 2022; Song et al., 2024), the present study focused on hybrid indica rice because it dominates high-yield production in southern China and is widely cultivated in tropical rice systems. Hybrid indica cultivars exhibit strong heterosis and high yield potential, but their genotypic divergence in nitrogen response under tropical hydrothermal conditions remains insufficiently characterized. Evaluating nitrogen-use performance within this group is therefore of practical significance for regional nutrient management strategies.

To minimize variability arising from heterosis and diverse genetic backgrounds, all cultivars were evaluated under the same site, season, planting density, and standardized agronomic management in a replicated split-plot design. Under such controlled experimental conditions, the observed differences primarily reflect cultivar-specific responses to the imposed nitrogen gradients rather than environmental heterogeneity.

The primary prerequisite for breeding nitrogen-efficient rice varieties is the existence of significant genotypic variation in response to nitrogen (N) supply. Our study revealed a highly significant interaction between genotype and N rate (G × N) for grain yield, indicating that the 28 tested hybrid rice varieties possess distinct strategies for N utilization. Based on the yield response curves, we categorized these varieties into four clusters: High Potential Type (Cluster 1), Late Responder Type (Cluster 2), Stable Type (Cluster 3), and Inhibition Type (Cluster 4). This classification result aligns with the findings of Fageria and Baligar (2005) and Zhang et al. (2020), who also observed that rice cultivars could be grouped into distinctive types based on their N sensitivity and efficiency. The robustness of this classification is further supported by the fact that the tested cultivars represent diverse yet widely cultivated hybrid indica backgrounds, enhancing the practical relevance of the identified response types for southern rice production systems.

However, a notable difference in our study is the identification of a significant proportion of “Stable Type” varieties (Cluster 3), which maintained high yields (>8.0 t ha^−^¹) even under a 30% reduction in N fertilizer. This contrasts with earlier studies where yield penalties under reduced N were more prevalent (Fageria and Baligar, 2005), suggesting that recent breeding efforts in hybrid rice may have inadvertently improved low-N tolerance through heterosis.

The divergent yield response patterns observed in this study may be associated with differences in nitrogen uptake, assimilation, and signaling pathways reported in previous literature. For example, earlier studies have shown that many indica rice varieties carry the *NRT1.1B*-indica allele, which is associated with enhanced nitrate uptake compared to japonica varieties (Hu et al., 2015). In addition, genes such as *OsNR2*, *OsGRF4*, and *OsTCP19* have been reported to influence nitrogen assimilation efficiency and low-nitrogen adaptation (Gao et al., 2019; Liu et al., 2021).

Although no molecular or transcriptomic analyses were conducted in the present study, the contrasting phenotypic responses among clusters are consistent with the possibility that genetic variation in nitrogen transport or regulatory pathways may contribute to these differences. For instance, cultivars classified as the “High Potential Type” or “Stable Type” may possess more efficient nitrogen utilization mechanisms, whereas the reduced performance of Cluster 2 under low-N conditions could reflect limited adaptation to nitrogen-deficient environments. Similarly, the yield decline observed in the “Inhibition Type” (Cluster 4) under high nitrogen supply may be related to imbalances in carbon–nitrogen coordination, as suggested in previous physiological studies (Chen et al., 2022b).

It should be emphasized that these interpretations are speculative and based on previously reported gene functions. Future studies integrating transcriptomic profiling, haplotype analysis, or candidate gene validation will be necessary to determine whether these molecular factors segregate among the four response types identified here.

### 4.2 Synergistic mechanism of yield components and the impact of environmental stress

Yield formation in rice is a function of source-sink relationships, which are heavily influenced by N supply. Our correlation results confirmed that effective panicle number (EP) and spikelet number per panicle (SNP) were the primary drivers of yield variation, explaining the superiority of Cluster 1 and Cluster 3. This is consistent with the classical theory that N application primarily increases yield by expanding the “sink size” (total spikelet number) (Peng et al., 2006). However, a common trade-off exists in rice breeding: increasing the panicle number often leads to a reduction in panicle size or grain weight. In our study, the elite varieties in Cluster 1 broke this trade-off, achieving simultaneous increases in EP and SNP. This phenomenon suggests that these varieties may possess a more efficient canopy architecture or higher vascular bundle capacity, allowing for sufficient assimilate transport to support a larger sink size without abortion (Chen et al., 2022a; Zhai et al., 2023).

An observation from this experiment was that the overall 1000-grain weight and seed setting rate in this experiment were slightly lower than the potential genetic ceiling of hybrid rice. This phenomenon needs to be discussed in the context of the tropical climatic conditions at the experimental site, particularly maximum temperatures during grain-filling reaching 35 °C (see Site Description), consistent with known thresholds for heat stress (Jiang et al., 2016). Previous research indicates that high temperature stress (Heat Stress) significantly shortens the grain-filling duration and inhibits starch synthase activity, leading to lower grain weight (Kim et al., 2011; Jiang et al., 2016).

### 4.3 NAE and the feasibility of reduction strategies

Improving NAE is the core goal of “Green Super Rice” cultivation. Our data showed that NAE varied significantly among clusters, with Cluster 3 maintaining high efficiency (17.30 kg kg^−^¹) at medium-low N levels. Comparing our results with global datasets, the NAE of Cluster 4 at high N (dropping to ~7.0 kg kg^−^¹) was significantly lower than the global average for effective management (Dobermann and Cassman, 2005), indicating severe fertilizer wastage. The mechanisms behind the high NAE in Cluster 3 likely involve both morphological and physiological adaptations. Morphologically, N-efficient varieties often possess steeper and deeper root architectures, allowing them to capture leached nitrogen in deep soil layers—a critical trait in tropical regions with high rainfall (Lynch, 2019; Wang et al., 2023). Physiologically, they may maintain higher activities of key enzymes like Glutamine Synthetase (GS) and Glutamate Synthase (GOGAT) under low N supply, ensuring that absorbed inorganic nitrogen is rapidly assimilated into amino acids for grain development (Liu et al., 2022b; Fortunato et al., 2023; Zhang et al., 2025a).

Based on the performance of the 10 screened elite varieties, our study supports the feasibility of a “30% N reduction strategy” under the experimental conditions tested. Specifically, reducing N application from 180 kg N ha^−^¹ to 120 kg N ha^−^¹ did not result in significant yield loss for Cluster 3 varieties. This finding suggests the potential for reducing nitrogen input through cultivar selection. Such reduction in fertilizer application may contribute to lowering nitrogen losses, including nitrate leaching and N_2_O emissions, as potential environmental co-benefits in intensive rice cropping systems (Zhang et al., 2018). However, these environmental outcomes were not directly measured in the present study and would depend on field-scale adoption and site-specific management practices.

From a practical production perspective, the four nitrogen-response types identified in this study provide differentiated management guidance. The “Stable Type” (Cluster 3), which maintained high yields under reduced nitrogen input, is particularly suitable for N-reduction programs and environmentally oriented production systems. The “High Potential Type” (Cluster 1) achieved superior yields under adequate N supply and may be preferred in high-input systems aiming to maximize productivity. In contrast, the “Late Responder Type” (Cluster 2), which showed weaker performance under low N conditions, may be less suitable for nitrogen-reduction strategies and requires sufficient N supply to express its yield potential. The “Inhibition Type” (Cluster 4) exhibited yield penalties under high N application and therefore should avoid excessive nitrogen input to prevent inefficiency and economic loss. Such classification-based management may help optimize fertilizer allocation according to varietal characteristics, thereby improving both agronomic and economic outcomes.

This study was conducted at a single tropical site and season; therefore, the identified nitrogen-response types and the feasibility of the 30% N reduction strategy should be interpreted within this environmental context. Given that nitrogen dynamics and yield formation may differ across rice-growing regions, multi-location and multi-season validation—including conventional temperate/subtropical regions—will be necessary to further assess the stability and generalizability of these response patterns.

It should be noted that only NAE was evaluated in this study. Future research incorporating plant nitrogen uptake measurements would enable calculation of additional NUE components, such as recovery efficiency and physiological efficiency, to provide a more comprehensive understanding of nitrogen-use mechanisms.

### 4.4 Towards sustainable rice production in tropical regions

While variety screening is the first step, achieving sustainable high yields in tropical rice-growing regions requires a holistic approach that integrates genetics with crop management (G × E × M). The tropical environment is characterized by high temperatures, frequent rainfall, and rapid soil organic matter decomposition, which poses unique challenges for N management. Firstly, Future Breeding Targets should shift from “High N Tolerance” to “Multi-Stress Tolerance”. As shown in our study, N deficiency often co-occurs with heat stress or transient drought in the tropics. Future breeding should utilize Genome-Wide Association Studies (GWAS) to identify gene modules that co-regulate N uptake and stress tolerance (e.g., *OsGRF4*) (Sun et al., 2020), aiming to create varieties that are robust against climate variability (Xin et al., 2025; Zhang et al., 2025b). The “Stable Type” varieties identified here serve as excellent donor parents for such programs.

Secondly, Integrated Nutrient Management (INM) is essential. In tropical regions, heavy rainfall leads to rapid leaching of urea. Simply reducing N rate might lead to deficiency in late growth stages if not managed correctly. Therefore, integrated nitrogen management should combine N−efficient varieties with appropriately selected enhanced−efficiency N fertilizers—such as slow/controlled−release formulations or urease/nitrification inhibitors—to better synchronize fertilizer N release with the crop N uptake curve, thereby improving nitrogen use efficiency, sustaining yields, and reducing N losses, provided that product type and N rate are optimized for the specific crop and environment (Folina et al., 2021). Additionally, incorporating organic amendments (biochar or manure) can improve the cation exchange capacity (CEC) of tropical soils, reduce N loss and enhance the sustainability of the system. Thirdly, Optimized Water Management, such as Alternate Wetting and Drying (AWD), should be explored in conjunction with these varieties. AWD can alter the soil redox potential and N form (ammonium vs. nitrate). Future research needs to investigate how the root systems of these “Stable Type” varieties respond to AWD cycles to maximize both water and nitrogen use efficiency.

## 5 Conclusion

This study evaluated the agronomic performance and nitrogen (N) response patterns of 28 indica hybrid rice cultivars in the tropical climate of Hainan. Our results demonstrated significant genotypic variation in yield responsiveness to N inputs, leading to the classification of these varieties into four distinct functional clusters: High Potential Type, Late Responder Type, Stable Type, and Inhibition Type. A key finding of this research is the identification of 10 elite cultivars (including V05, V07, and V26) that achieved stable yields exceeding 8.0 t ha^−^¹ under a medium-low nitrogen rate (120 kg N ha^−^¹). This represents a 33% reduction in N fertilizer compared to the local standard practice (180 kg N ha^−^¹) without a significant compromise in productivity. The physiological basis for this N-efficient and stable-yielding performance was primarily attributed to the high plasticity of effective panicle number per hill and spikelet number per panicle. Overall, these findings provide a scientific foundation for the selection and breeding of N-efficient varieties specifically adapted to tropical agroecosystems. Promoting these “Stable Type” cultivars offers a practical pathway to decouple grain yield from excessive nitrogen dependence, thereby enhancing the sustainability of rice production in the tropics.

## Data Availability

The original contributions presented in the study are included in the article/supplementary material. Further inquiries can be directed to the corresponding authors.
